# SapC-DOPS-induced lysosomal cell death synergizes with TMZ in glioblastoma

**DOI:** 10.18632/oncotarget.2232

**Published:** 2014-07-18

**Authors:** Jeffrey Wojton, Walter Hans Meisen, Naduparambil K. Jacob, Amy Haseley Thorne, Jayson Hardcastle, Nicholas Denton, Zhengtao Chu, Nina Dmitrieva, Rachel Marsh, Erwin G. Van Meir, Chang-Hyuk Kwon, Arnab Chakravarti, Xiaoyang Qi, Balveen Kaur

**Affiliations:** ^1^ Department of Neurosurgery, The Ohio State University Medical Center, Columbus, OH; ^2^ Department of Radiation-Oncology, The Ohio State University Medical Center, Columbus, OH; ^3^ Solid-Tumor Program at the James Comprehensive Cancer Center, The Ohio State University Medical Center, Columbus, OH; ^4^ Ludwig Institute for Cancer Research, University of California San Diego, La Jolla, California; ^5^ Departments of Medical Oncology and Molecular Medicine, Mayo Clinic, Rochester, MN; ^6^ The Vontz Center for Molecular Studies, Division of Hematology/Oncology, Department of Internal Medicine, University of Cincinnati College of Medicine, Cincinnati, OH; ^7^ Department of Neurosurgery and Hematology and Medical Oncology, Winship Cancer Institute and School of Medicine, Emory University School of Medicine, Atlanta, GA

**Keywords:** SapC-DOPS, glioblastoma, lysosomal dysfunction, TMZ, synergy

## Abstract

SapC-DOPS is a novel nanotherapeutic that has been shown to target and induce cell death in a variety of cancers, including glioblastoma (GBM). GBM is a primary brain tumor known to frequently demonstrate resistance to apoptosis-inducing therapeutics. Here we explore the mode of action for SapC-DOPS in GBM, a treatment being developed by Bexion Pharmaceuticals for clinical testing in patients. SapC-DOPS treatment was observed to induce lysosomal dysfunction of GBM cells characterized by decreased glycosylation of LAMP1 and altered proteolytic processing of cathepsin D independent of apoptosis and autophagic cell death. We observed that SapC-DOPS induced lysosomal membrane permeability (LMP) as shown by LysoTracker Red and Acridine Orange staining along with an increase of sphingosine, a known inducer of LMP. Additionally, SapC-DOPS displayed strong synergistic interactions with the apoptosis-inducing agent TMZ. Collectively our data suggest that SapC-DOPS induces lysosomal cell death in GBM cells, providing a new approach for treating tumors resistant to traditional apoptosis-inducing agents.

## INTRODUCTION

Gliomas constitute nearly 70% of all malignant primary brain tumors[1] and have been well-characterized for their inherent and acquired resistance to chemotherapy and radiotherapy.[2, 3] Despite surgical resection, chemotherapy with temozolomide (TMZ), and radiotherapy median survival of patients diagnosed with a glioblastoma (GBM) is less than 15 months.[4, 5] Part of the recalcitrant nature of these tumors is due to their intrinsic resistance to therapy-induced apoptosis and enhanced survival signaling.[6] There is therefore an unmet need for novel therapeutics and treatment strategies to combat this disease.

SapC-DOPS are stable nanovesicles formed by the coupling of the sphingolipid activating protein saposin C (SapC) and dioleoylphosphatidylserine (DOPS).[7] Recently we demonstrated the ability of systemically administered SapC-DOPS nanovesicles to specifically target brain tumors and the tumor-associated vasculature resulting in significant antitumor effects.[8] Treatment of neuroblastoma and pancreatic cancer cells with SapC-DOPS has been shown to generate increased ceramide levels through activation of acid sphingomyelinase, leading to subsequent caspase activation and apoptosis.[7, 9] However, most GBM cells are intrinsically resistant to apoptosis and the mechanism of cell death induced by SapC-DOPS in GBM remains to be elucidated. In this study we examined the activation of key downstream molecular signaling events following SapC-DOPS treatment in GBM and report a novel mechanism of action for SapC-DOPS-induced cell death, which can be combined with TMZ to improve efficacy.

## RESULTS/DISCUSSION

### SapC-DOPS-induced cell death is independent of apoptotic and autophagic cell death

**Figure 1:**
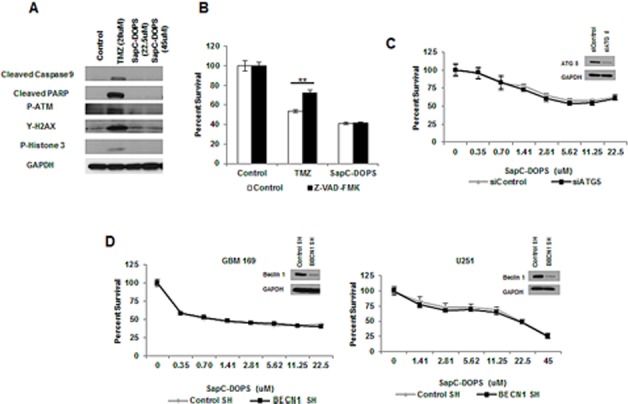
SapC-DOPS-induced cell death is independent of apoptotic and autophagic cell death (A) Apoptotic and DNA damage markers were analyzed in GBM 169 neurospheres by immunoblot 48h following treatment with TMZ or SapC-DOPS (indicated doses). (B) GBM 169 neurospheres were treated with TMZ (20μM) or SapC-DOPS (22.5μM) with or without Z-VAD-FMK (100μM) and cell viability was determined 5 days later. (C) Cell viability of GBM 169 neurospheres transiently knocked down for ATG5 and treated with SapC-DOPS for 5 days. (D) Cell viability for GBM neurospheres and U251 GBM cells with stable knock down of BECN1 treated with SapC-DOPS for 5 days. Viability was measured using MTT, error bars show mean + SD.

Previously we observed SapC-DOPS treatment of neuroblastoma and pancreatic cancer cells to induce caspase activation and apoptosis;[7, 9] however, western blot analysis of primary GBM neurospheres or serum-cultured GBM cells treated with LD_50_ doses of SapC-DOPS revealed no obvious change in caspase activation or DNA damage markers (Fig. 1A and Supplemental Fig. 1A). Consistent with this SapC-DOPS treatment did not induce cell cycle arrest and was not dependent on p53 status (Supplemental Fig. 1B-D). Additionally, treatment of GBM neurospheres with the pan-caspase inhibitor Z-VAD-FMK did not rescue SapC-DOPS-induced cytotoxicity, suggesting a caspase-independent mechanism of cell death (Fig. 1B). To determine whether this lack of apoptotic signaling was specific to GBM, we treated a panel of cancer cell lines with SapC-DOPS and evaluated apoptotic markers via western blot. Interestingly, we observed increased levels of cleaved PARP and/or γ-H2AX in 5/8 of these cell lines, suggesting that the mechanism by which SapC-DOPS kills GBM cells may depend on the genetic/molecular alterations within the cell and future studies will determine the key signaling pathways that govern the mechanism of cell death execution in different cells (Supplemental Fig. 2A-C). Morphological characterization of SapC-DOPS-induced cell death by transmission electron micrography revealed a lack of morphological hallmarks of apoptosis (chromatin condensation and nuclear fragmentation), but did reveal cytoplasmic vacuoles with features of autophagosomes (Supplemental Fig. 3A-B). While induction of LC3-II is observed in GBM cells treated with SapC-DOPS, knockdown of autophagy dependent genes ATG5 and BECN1, which reduced LC3-II levels (Supplemental Fig. 3C), had no effect on SapC-DOPS-induced cytotoxicity (Fig. 1C-D). Future studies will be necessary to identify the importance of autophagy signaling in response to SapC-DOPS treatment *in vivo*.

### SapC-DOPS induces lysosomal cell death via membrane permeability and organelle dysfunction

**Figure 2:**
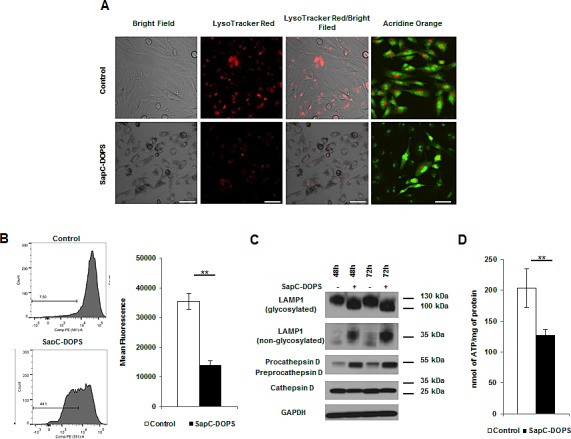
SapC-DOPS induces lysosomal cell death via membrane permeability and organelle dysfunction (A) Lysosomes were analyzed by fluorescent microscopy in U251 GBM cells treated with SapC-DOPS (45μM) using LTR or AO at 48h (B) or by FACS analysis using LTR. Numbers indicate the percentage of cells with decreased fluorescence. (C) Lysosomal proteins were analyzed via immunoblot from U251 GBM cells treated with SapC-DOPS (45μM). (D) ATP was measured in U251 GBM cells 48h following treatment with SapC-DOPS (45μM). Error bars show mean + SD, **P<.01. Scale bar 100μm.

Saposins are localized to the lysosome where they activate various lysosomal lipid degrading enzymes. [10] To test whether SapC-DOPS affected lysosome function/integrity we investigated the impact of SapC-DOPS on lysosomes using the lysosomotropic fluorochrome Acridine Orange (AO) and the acidophilic dye LysoTracker Red (LTR). Treatment with SapC-DOPS resulted in a substantial decrease in acidic vesicular organelles as determined by decreased red fluorescence of AO and LTR (Fig. 2A). Quantification of LTR uptake by FACS analysis also revealed a significant reduction in LTR fluorescence following treatment implicating an impact of SapC-DOPS on lysosome number and/or integrity (Fig. 2B). Lysosomal-associated membrane protein 1 (LAMP1) is an extensively glycosylated transmembrane protein which protects the lysosome from self-destruction whose size varies from approximately 130 kDa (glycosylated) to 30 kDa (unglycosylated).[11] LAMP1 expression and/or changes in its glycosylation state reflect lysosomal stability and function. Western blot analysis revealed no change in total LAMP1 protein levels, but did reveal a reduction in the levels of glycosylation of LAMP1, evident by its faster migration (100 vs. 130 kDa) concomitant with an increase in the unglycosylated (30 kDa) form (Fig. 2C and Supplemental Fig. 4A). Cathepsin D is the most abundant lysosomal aspartate protease, which is synthesized at 52 kDa (procathepsin D), targeted to the lysosome by the mannose-6-phosphate receptor and cleaved yielding a 48 kDA fragment (preprocathepsin D) that is further processed by lysosomal proteases into the mature 34 kDa fragment.[12] SapC-DOPS treatment revealed a reduction in the levels of mature cathepsin D, with a concomitant increase in the pro/preprocathepsin D levels (Fig. 2C and Supplemental Fig. 4A). Changes in LMP and alterations in the glycosylation of LAMP1 and proteolytic processing of cathepsin D indicate significant lysosome dysfunction induced by SapC-DOPS. Furthermore, we observed a significant decrease in ATP levels following SapC-DOPS treatment indicative of necrosis (Fig. 2D and Supplemental Fig. 4B). Interestingly, almost all cancer cell lines analyzed displayed similar alterations to LAMP-1 (Supplemental Fig. 2A-C) irrespective of the induction of markers of apoptosis, indicating that susceptibility of different cell types to SapC-DOPS-induced lysosomal damage may be a key mechanism involved in SapC-DOPS-induced cytotoxicity.

### SapC-DOPS alters sphingolipid intermediates

**Figure 3:**
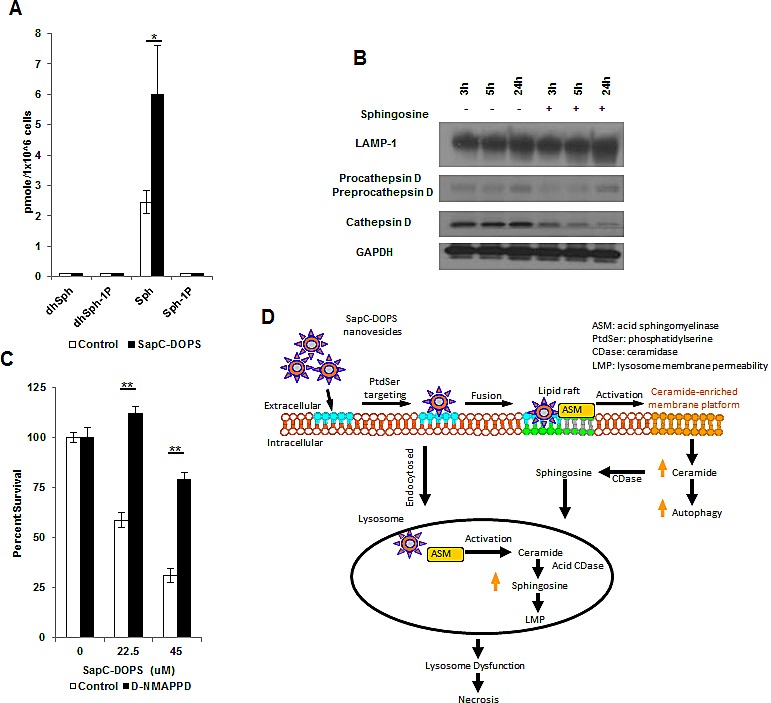
SapC-DOPS alters sphingolipid intermediates (A) GBM 169 neurospheres were treated with SapC-DOPS (45μM) for 48h and analyzed for sphingosine species. (B) GBM 169 neurospheres were treated with sphingosine (10μM) or DMSO and lysosomal markers were analyzed via immunoblot. (C) U251 GBM cells were treated with SapC-DOPS with or without D-NMAPPD (1μM), cell viability was measured 5 days later by MTT. Error bars show mean + SD, **P<.01, *P<.05. (D) Hypothetical summary figure of SapC-DOPS in GBM.

Alterations in the balance of bioactive sphingolipids/ceramides/sphingosine-1 phosphate have been shown to regulate pro-survival and pro-death signaling in numerous tumor types including GBM.[13, 14] We investigated changes in sphingolipid intermediates through an unbiased liquid chromatography-mass spectrometry lipid analysis (Supplemental Fig. 5A-B) Sphingosine, which has been shown to be a potent inducer of apoptotic and necrotic cell death through LMP[15], was observed to increase significantly following SapC-DOPS treatment, with no detectable increase in sphingosine-1 phosphate levels (Fig. 3A). Next we treated the GBM neurospheres with sphingosine (10 μM) and observed a decrease in the mature form of cathepsin D and a slightly faster migration of LAMP-1 resembling the responses observed after SapC-DOPS treatment (Fig. 3B). Interestingly, sphingosine has been shown to induce Golgi fragmentation, which could be responsible for the decrease in global glycosylation and future studies will reveal the exact mechanism for SapC-DOPS-induced LAMP1 changes.[16, 17] Pre-treating GBM neurospheres with the acid ceramidase inhibitor D-NMAPPD, preventing the hydrolysis of ceramide into sphingosine, resulted in a significant rescue of cell viability following SapC-DOPS (Fig. 3C). We propose that SapC-DOPS treatment leads to the catabolism of sphingomyelin into ceramide, which is converted to sphingosine, resulting in LMP and lysosome dysfunction-induced necrotic cell death. It is possible that the levels of ceramide synthase, ceramidase, and sphingosine kinase in different cell types may dictate how they respond to SapC-DOPS treatment, resulting in increased ceramide and corresponding apoptosis in some cells and increased sphingosine, LMP, and necrotic cell death in others.

### SapC-DOPS and TMZ displays synergistic interactions in combination

**Figure 4:**
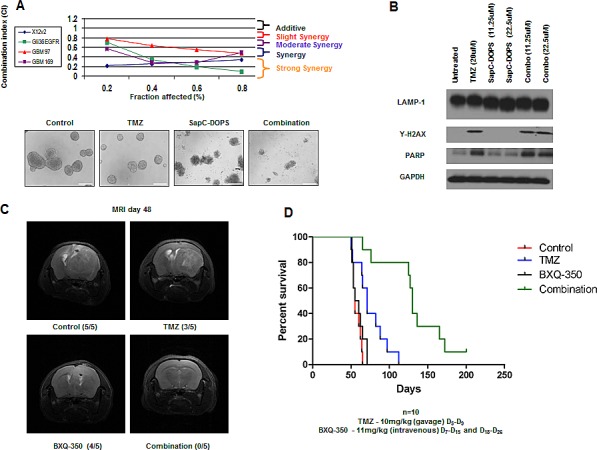
SapC-DOPS and TMZ yields synergistic interactions in combination (A) Chou-Talalay analysis of the combination of SapC-DOPS and TMZ. Data is shown as fraction affected (fa) versus combination index (CI) plots. CI < 1 indicates synergy, CI=1 indicates additive, and CI > 1 indicates antagonistic interactions. Representative images of GBM neurospheres treated with TMZ (20μM), SapC-DOPS (45μM), combination, or vehicle for 5 days. (B) GBM 169 neurospheres were treated with TMZ, SapC-DOPS, or the combination for 48h and evaluated for lysosomal and apoptotic markers. (C) Representative T2-weighted MRI images of coronal sections of mice with GBM 169 neurospheres intracranial tumors treated with PBS, TMZ (10mg/kg) D5-D9, BXQ-350 (11mg/kg) D7-D15 and D18-D26, or the combination. Scans were performed 48 days from tumor implantation. (D) Scale bar 100μm. Kaplan-Meir survival curve for C, n=10, P=.0003.

Based on the caspase-independent signaling in GBM following SapC-DOPS treatment, we investigated possible synergistic interactions in combination with the apoptosis-inducing agent TMZ. Moderate to strong synergy was observed using the median-effect method of the Chou-Talalay analysis in a panel of traditional serum-cultured GBM cells (X12v2 and Gli36ΔEGFR) and GBM neurospheres (GBM 97 and GBM 169) (Fig. 4A). Immunoblots of GBM neurospheres revealed a dose-dependent reduction in the size of LAMP1 in neurospheres treated with SapC-DOPS alone and increased y-H2AX in neurospheres treated with TMZ alone and the induction of both pathways in neurospheres treated with TMZ and SapC-DOPS (Fig. 4B). In order to evaluate the effect of TMZ and SapC-DOPS combination *in vivo*, we established intracranial GBM 169 xenografts in athymic nude mice and treated them with systemically administered BXQ-350, the clinical formulation of SapC-DOPS, TMZ, or the combination. Tumor presence and size was evaluated by T2-weighted MRI in a random selection of 5 mice/group and visible tumors were observed in 5/5 control mice, 3/5 TMZ treated mice, 4/5 BXQ-350 treated mice, and 0/5 combination treated mice (Fig. 4C). None of the mice treated with the combination of TMZ and BXQ-350 displayed any visible signs of tumor 48d post tumor implant and there was a significant enhancement in median survival of mice compared to either agent alone (Fig. 4D). The majority of the mice do eventually succumb to disease, which could be a result of a small set of tumor cells that are inherently resistant to this treatment or residual tumor cells may have acquired resistance through additional mutations and/or activation of compensatory survival pathways.

## MATERIAL AND METHODS

See Supplemental Material and Methods

### Cell Culture and Reagents

Human U251, CAL 27, SCC-74A, MDA-231, SKOV-3, PC-3, H446, and H1299 cell lines were obtained from ATCC. CHLA-20 cells were provided by Thomas Inge, LNZ308-2024 p53 tet-on and control LNZ308-C16 human GBM cells were kindly provided by Erwin G. Van Meir [18], and X12 cells were obtained from Dr Sarkaria and subcloned to express GFP to generate X12v2 (Mayo Clinic, Rochester, MN). GBM cells and GBM neurospheres were cultured as described (Supplemental Materials/Methods). For cytotoxicity assays the indicated cells were plated in 96-well dishes and treated with SapC-DOPS for 5 days. Cell viability and ATP quantification was determined by MTT assay (Roche), and ATP assay kit (Abcam) respectively according to the manufacturer’s instructions. SapC-DOPS was formulated as described.[7-9]

### Lipid Quantifications

Sphingolipids were measured using liquid chromatography-mass spectrometry method at the lipodomics core of the Medical University of South Carolina as described[19].

### *In Vivo* Xenografts

Anesthetized mice fixed in a stereotactic apparatus were implanted at 2 mm lateral to bregma, at a depth of 3 mm with GBM 169 cells (1×10^5^). Mice were treated with TMZ/DMSO by gavage and BXQ-350/DOPS by tail vein injection, at doses/days indicated.

### Statistical Analysis

Student’s t-test was used to analyze *in vitro* experiments. P<0.05 was considered statistically significant in Student’s t-test and all error bars represent standard deviation. See supplemental materials and methods.

## SUPPLEMENTARY FIGURES


