# Supplementary material for: SapC-DOPS-induced lysosomal cell death synergizes with TMZ in glioblastoma
Source: Oncotarget. 2014 Jul 18;5(20):9703–9. doi: 10.18632/oncotarget.2232 (PMC4259431; doi:10.18632/oncotarget.2232)
Supplement: Supplementary file 1 [file oncotarget-05-9703-s001.pdf]

SapC-DOPS-induced lysosomal cell death synergizes with TMZ in glioblastoma

Supplementary Materials and Methods

Cell Culture

Cells were cultured as follows: U251, CAL 27, SCC-74A and MDA-231 cells in DMEM supplemented with 10% fetal bovine serum, SKOV-3 cells in MEM with 20% fetal bovine serum, PC-3, H466, and H1299 cells in RPMI with 10% fetal bovine serum, and CHLA-20 cell in 20% IMDM with 20% fetal bovine serum. All cells were cultured in 100 units of penicillin/ml and 10 mg of streptomycin/ml. GBM 97, and 169 were obtained under an approved Institutional Review Board (IRB) protocol from the office of Responsible Research Practices at The Ohio State University Medical Center where written consent for the collection of surgical specimens was obtained from patients, as described.[1] Patient-derived GBM neurospheres were isolated and maintained as tumor spheres in Neurobasal Medium supplemented with 2% B27, human EGF (50 ng/ml), and bFGF (50 ng/ml) and penicillin-streptomycin in low-attachment cell culture flasks as described.[1] All cells were routinely checked for mycoplasma contamination. Temozolomide (Sigma), D-Sphingosine (Sigma), Acridine Orange (Sigma), D-NMAPPD (Cayman), Z-VAD-FMK (Promega), and LysoTracker Red (Invitrogen) were used according to manufacturer's instructions.

Western Blot Analysis

Protein concentrations were determined by Pierce BCA protein assay kit (ThermoFisher) and equal amounts of protein were separated on a 4-20% Tris-HCL gel and transferred to a PVDF membrane. Primary antibodies P-Histone 3 (Millipore) and all other antibodies (Cell Signaling) were used at 1:1000. GAPDH (Millipore) was used as a loading control.

Flow Cytometry

For cell cycle analysis, cells were centrifuged, washed with PBS and fixed in EtOH and stained with propidium iodide (50ug/ml) in PBS with RNase A (10ug/ml). Samples were analyzed in a FACS Calibur (Becton Dickinson). For LMP quantification, cells were treated with 75nM of LysoTracker Red for 30 min at 37*C and analyzed in a FACS Aria III (Becton Dickinson). At least 10,000 events were used for evaluation and samples were run in triplicate.

Transfections

Transient siRNA knockdown experiments were completed by transfecting cells with On-Target plus SMART pool ATG5 (Dharmacon) using RNAi Max lipofectamine (Invitrogen) per manufacturer's instructions. Stable knockdown of BECN1 and control SH were established using lentiviral particles (Santa Cruz) per the manufacturer's instructions.

Imaging

Immunofluorescence: For detection of acidic vacuoles, cells were treated with acridine orange at a final concentration of 1 µg/ml or LysoTracker Red at 100nM for 30min. Images were taken using an inverted fluorescent microscope Olympus (U-TB190) or Zeiss LSM 510 Meta confocal microscope. Electron Microscopy: GBM 169 neurospheres were treated with SapC-DOPS for 48 hours, washed with PBS, and fixed with 2.5% glutaraldehyde in .1M phosphate buffer, pH 7.4 containing .1M sucrose, washed with PBS, and resuspended in 2% agarose. Cells were then post-fixed in OsO_4, stained with uranyl acetate and embedded in Epon resin and viewed with a FEI Tecnai G2 Spirit. Magnetic Resonance Imaging: Anatomic imaging was done on 48 days

following tumor implantation using T2-weighted RARE imaging sequence (TR=2500 ms, TE=12 ms, Rare Factor=8, navgs=4). The imaging was performed using a Bruker Biospin 94/30 magnet (Bruker Biospin, Karlsruhe Germany). The acquisition parameters for T2-weighted multi-slice scans were as follows: FOV = 20 mm x 20 mm, slice thickness = 1.0 mm, matrix size = 256 x 256.

Statistical Analysis

For *in vivo* survival studies Kaplan–Meier curves were compared using the logrank test using GraphPad Prism S/W. Chou-Talalay synergy analysis was completed as follows: For constant ratio Chou-Talalay analysis, the median dose (Dm) for TMZ and SapC-DOPS was determined from dose response curves from each agent alone using CompuSyn software and the law of mass action: log(fa/fu)=(m)logD-(m)logDm. Fraction affected (fa), fraction unaffected (fu), dose (D), median dose (Dm), coefficient for shape of the dose-response curve (m). Individual Dm values were obtained for TMZ and SapC-DOPS in each cell line or GBM neurosphere culture, and respective Dm for each agent was added in combination and serially diluted in a constant ratio and added to cells for combination treatment. Individual treatments were repeated with the drug preps from combination treatments. Combination dose-response curves were fit to Chou-Talalay lines and combination indexes (CIs) were calculated for each fraction affected (Fa) using the CompuSyn software and the following equation $CI=(D_1/Dx_1) + (D_2/Dx_2) + (D_1)(D_2)/[(Dx_1)(Dx_2)]$. Dx_1 and Dx_2 are the TMZ and SapC-DOPS doses that are required to achieve a Fa and D_1 and D_2 are the doses of TMZ and SapC-DOPS in combination required to achieve the Fa. CI values represent antagonism >1.1, additive 0.9 to 1.1, slight synergy 0.8 to 0.9, moderate synergy 0.6 to 0.8, synergy 0.4 to 0.6, and strong synergy < 0.4. [2, 3]

References

1. Otsuki A, Patel A, Kasai K, Suzuki M, Kurozumi K, Chiocca EA and Saeki Y. Histone deacetylase inhibitors augment antitumor efficacy of herpes-based oncolytic viruses. Molecular therapy : the journal of the American Society of Gene Therapy. 2008; 16(9):1546-1555.
2. Chou TC. Theoretical basis, experimental design, and computerized simulation of synergism and antagonism in drug combination studies. Pharmacological reviews. 2006; 58(3):621-681.
3. Chou TC. Drug combination studies and their synergy quantification using the Chou-Talalay method. Cancer research. 2010; 70(2):440-446.

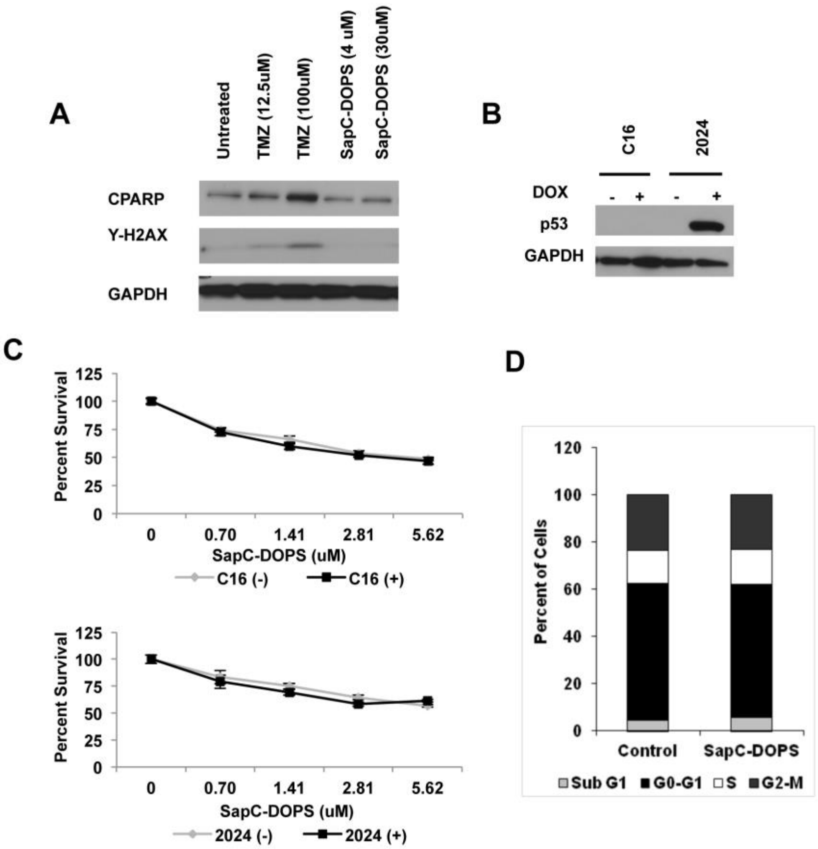

Fig. 1: (**A**) Immunoblot of X12v2 GBM cells treated with TMZ or SapC-DOPS at indicated doses for 48h. (**B**) Immunoblot of p53-null clone 2024 with dox-inducible wt-p53 expression and parental control 24h following doxycycline treatments (2μg/ml) (see Materials and Methods). (**C**) Cells were treated with doxycycline (2μg/ml) for 24h and then treated with SapC-DOPS, cell viability was measured 5 days later. (**D**) Cell cycle analysis of GBM 169 neurospheres 48 hours following treatment with SapC-DOPS (45μM).

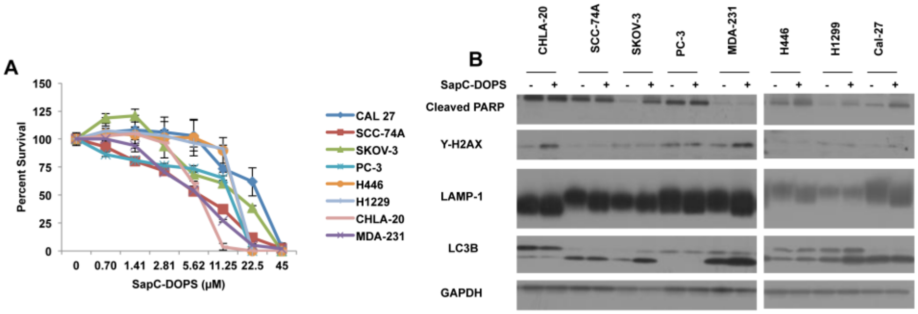

C

Cell Line	p53 Status	Cancer Type	SapC-DOPS (μM)	PARP cleavage	DNA Damage	LAMP-1 size shift	Autophagy Induction
CHLA-20	Wild-Type	Neuroblastoma	5.63	-	+	-	-
SKOV-3	Mutant	Ovarian	22.5	+	-	-	+
PC-3	Null	Prostate	11.25	-	-	-	-
MDA-231	Mutant	Breast	16.88	-	+	+	+
H446	Mutant	SCLC	16.88	+	-	+	-
H1299	Null	NSCLC	16.88	+	-	-	+
CAL-27	Mutant	Squamous cell carcinoma	22.5	+	-	+	-
SCC-74A	Wild-Type	Squamous cell carcinoma	16.88	-	-	+	-

Fig. 2: (**A**) A panel of cancer cell lines were treated with SapC-DOPS at indicated doses, viability was measured 5 days later by MTT, error bars show mean ± SD. (**B**) Apoptosis, autophagy, and lysosomal proteins were analyzed by western blot from indicated cell lines 48 hours following treatment. (**C**) Table indicating the cancer cell type and doses used for western blot analysis in **B**.

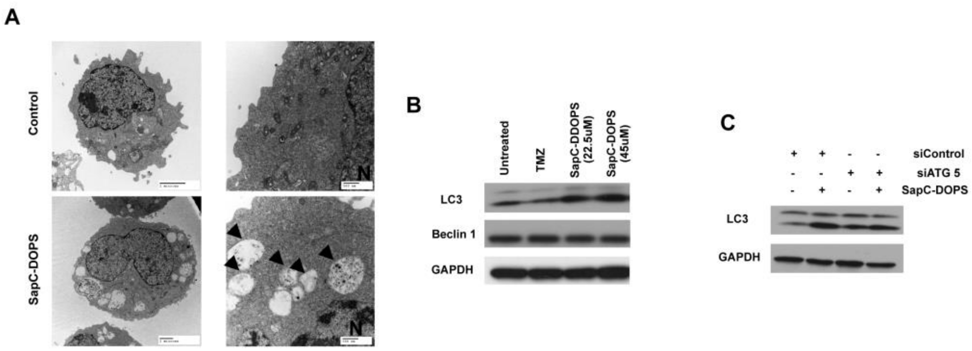

Fig. 3: (**A**) Transmission electron microscopy of GBM 169 neurospheres treated with SapC-DOPS (45μM) for 48h. Representative images are shown, arrowheads point to autophagosomes and the nucleus (N) is labeled. (**B**) Immunoblot of GBM 169 neurospheres treated with TMZ (20μM) or SapC-DOPS (22.5μM) for 48h. (**C**) Immunoblot of GBM 169 knockdown neurospheres treated with SapC-DOPS for 72h. Scale bar 500nm.

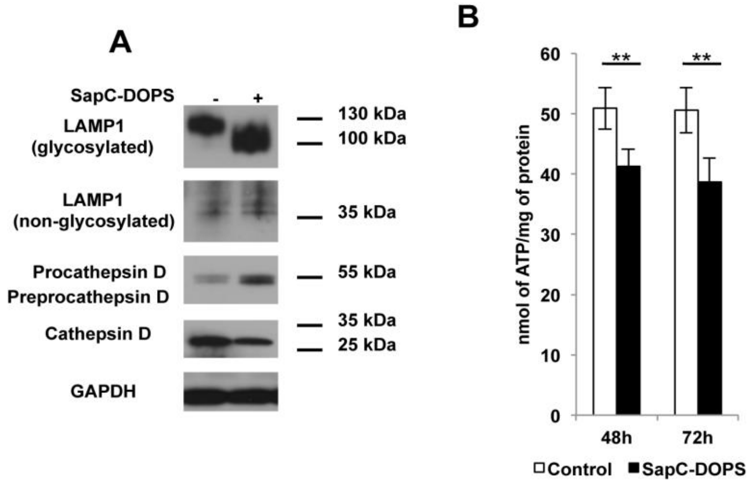

Fig. 4: (**A**) Lysosomal proteins were analyzed via immunoblot from GBM 169 neurospheres treated with SapC-DOPS (45µM) for 72h. (**B**) ATP was measured in GBM 169 neurospheres following treatment with SapC-DOPS (45µM). Error bars show mean ± SD, **P<.01.

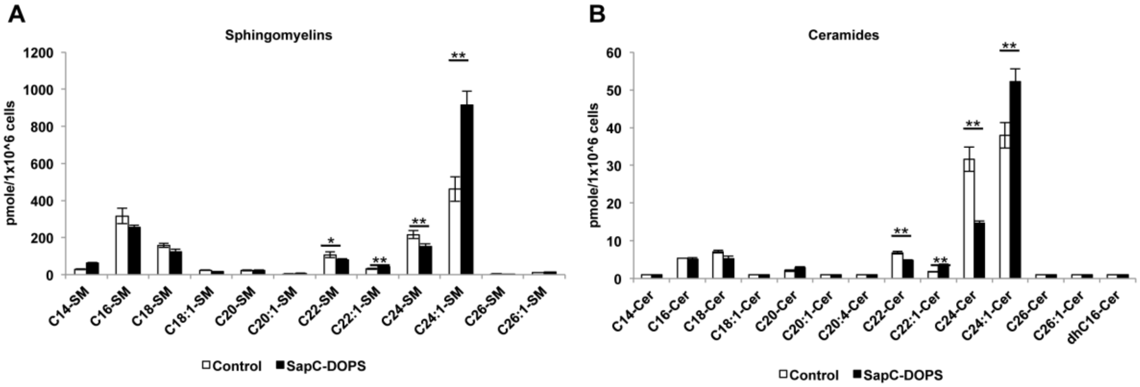

Fig. 5: GBM 169 neurospheres were treated with SapC-DOPS (45μM) for 48h and analyzed for (**A**) sphingomyelin and (**B**) ceramide species.
